# Use and knowledge of the razor-billed curassow *pauxi tuberosa *(spix, 1825) (galliformes, cracidae) by a riverine community of the oriental amazonia, brazil

**DOI:** 10.1186/1746-4269-7-1

**Published:** 2011-01-02

**Authors:** Flávio B Barros, Henrique M Pereira, Luís Vicente

**Affiliations:** 1 Universidade Federal do Pará, Campus Universitário de Altamira, Faculdade de Educação, Rua Coronel José Porfírio, 2515, São Sebastião 68372-040, Altamira, Pará, Brazil; 2 Centro de Biologia Ambiental, Faculdade de Ciências da Universidade de Lisboa, Campo Grande, 1749-016 Lisboa, Portugal

## Abstract

In the Amazonian basin, the human populations that traditionally inhabit the forest use its natural resources in various ways. One example is the local fauna which, among several other uses, is an important source of protein. The general aim of our study was to investigate the importance of hunting to the lives of the Amazonian riverine communities and to identify the multiple uses and knowledge about the hunted animals. In this article we focused the study on the razor-billed curassow *Pauxi tuberosa*, a Cracidae of significant value to the studied community. The investigation was conducted in the "Riozinho do Anfrísio Extractive Reserve", a Brazilian Conservation Unit located at the Altamira municipality, in the state of Pará. We used an ethnoecological approach, which included participant observation and semi-structured interviews. Our results show that the razor-billed curassow is used by the "Riozinho do Anfrísio" local population mainly as food, but it also fulfils secondary functions, with the feathers being used as a domestic tool and as magic-religious symbol, some organs as traditional medicine, and some chicks even being raised as pets. Our study also revealed that the traditional ecological knowledge of the riverines about their environment is considerably large, and that the local biodiversity provides various ecosystem services.

## Introduction

Animals have long been used by humans for the most diverse purposes. In the last decade, however, the different ways in which the faunistic resources are used by traditional human cultures have become a significant subject of investigation in Brazil [1-10] and other countries [11-14], due to their overall importance to conservation issues. Some important uses include: food, zootherapy, pet rearing (***xerimbabismo***), ornamentation, manufacturing of domestic tools and magic-religious symbolism [15,3,7,20]. To a significant part of the Brazilian population, and in particular to the Amazonian riverine communities, animal resources represent an important source of protein and traditional medicine, since these populations are isolated and thus depend primarily on the natural resources obtained directly from the forest. Hunting is the most common strategy used by the Amazonian riverines to obtain animal resources and several studies have been discussing the various aspects of this practice, namely its inventory, characterization [8] and impact on the animal populations [4,5,21,22], as well as other related subjects [10]. The aim of the present study was to describe the hunting activities and the uses and knowledge of the local fauna by a riverine community of the Oriental Amazonia.

In Amazonia, the most hunted bird species are those from the Cracidae family [11-13,23]. Cracidae birds occur exclusively in the American continent, from Mexico to Argentina [12]. The family includes fifty large bird species that inhabit tropical and subtropical forests, with few species being found in open areas [24]. It is the most threatened bird family of the Americas, mostly due to habitat destruction and hunting [25,26]. The majority of the targeted species have life history traits incompatible with intensive hunting: they are monogamous and only rear one brood of one to three eggs per year. Hatchlings suffer high mortality for the first year of life and only reach maturity after the third year [26,27]. These birds are considered important bioindicators of the ecosystems' health because they need large breeding territories and are major seed dispersers, acting as restorative agents of the tropical forest ecosystems [25,26,28,29]. Yet, in many American regions, over-hunting caused the decrease of various Cracidae populations and the local extinction of some species [12,30].

We focus the present study in the razor-billed curassow, *Pauxi tuberosa *(locally known as ***mutum-fava***), which according to the Cracidae specialists group of the IUCN [31] is of "intermediate conservation priority". *Mitu tuberosa*, *Mitu tuberosum *and *Crax tuberosa *are synonyms [31] of *P. tuberosa *[32]. The species distribution range includes Brazil, Peru, Colombia and Bolivia [31]. It is described as uncommon, but its status in the IUCN red list is that of "least concern" [31]. However, according to Ohl-Schacherer et al. [14] and Begazo [27], *P. tuberosa *is a quite commonly hunted Cracidae in the Amazonia, and there seems to be, based on the available literature, a lack of studies on the biology and ecology of the species. Muñoz & Kattan [28] argued that the major limitation for the development of conservation plans to Cracidae species is the lack of knowledge on fundamental aspects of the family behavioural ecology, such as the type of habitats that the birds prefer, how they interact with each other, and how they establish territories among the chosen patches.

## Materials and methods

### Study Area and Community

**Figure 1:**
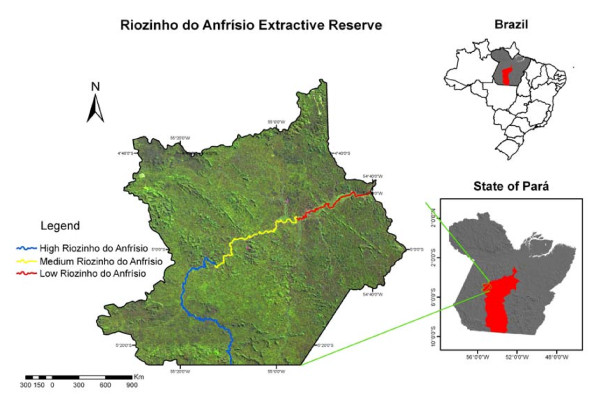
**Localization of the "Riozinho do Anfrísio Extractive Reserve": Altamira municipality, southwest Pará state, northern Brazil**.

The present study was conducted in a conservation unit named "Reserva Extrativista Riozinho do Anfrísio" (54°39'18.28"W, 4°45'33.98"S). According to the Brazilian National System of Conservation Units [33], "Reserva Extrativista" (which stands for "Extractive Reserve") is a conservation unit in the category of "sustainable use", since it allows the presence of human populations within the reserve, as well as the sustainable exploitation of its natural resources. The territorial size of this extractive reserve is of 736 340 ha and is located at the Altamira municipality, in the state of Pará, northern Brazil (Figure 1). The Altamira municipality is located at the southwest of the Pará state, in the Trans-Amazonian and Xingu region. Altamira has a surface of 159 695.94 km^2 ^and a population of 98.750 people, the majority of which is living in urban areas [34]. The territory that comprises the conservation unit is known as the "Terra do Meio", because the region includes the lands that are located between the Rivers Xingu and Tapajós [35]. The reserve was implemented in 2004 by the Brazilian Ministry of Environment with the aim of preserving its natural resources and of protecting the traditional populations' lifestyle [36]. It was primarily established to fight illegal lumberers and land-grabbers (***grileiros***) of the Brazilian State, since they were threatening both the forest and the permanence of the traditional populations in the territory.

The study area is characterized by a hot and humid climate, with mean annual temperature of 27°C, and mean annual rainfall of 1.885 mm. The vegetation is of the tropical moist broadleaf forest type, but rather opened and mixed. The region is alluvial and submontane [37].

The majority of the local population is illiterate, and until recently almost nobody owned a birth certificate or another civil document. There is no medical assistance, since there are no doctors, nurses and health centres [38]. In the study area there are, at present, 56 families, representing a total of 290 people, from which the majority are children and youngsters. The colonization of this Amazonian region happened at the beginning of the XX^th ^century, at the caoutchouc epoch. This had lead to the mixing of the indigenous population with the migrant people, which had come from other Amazonian regions and from Northeastern Brazil.

**Figure 2:**
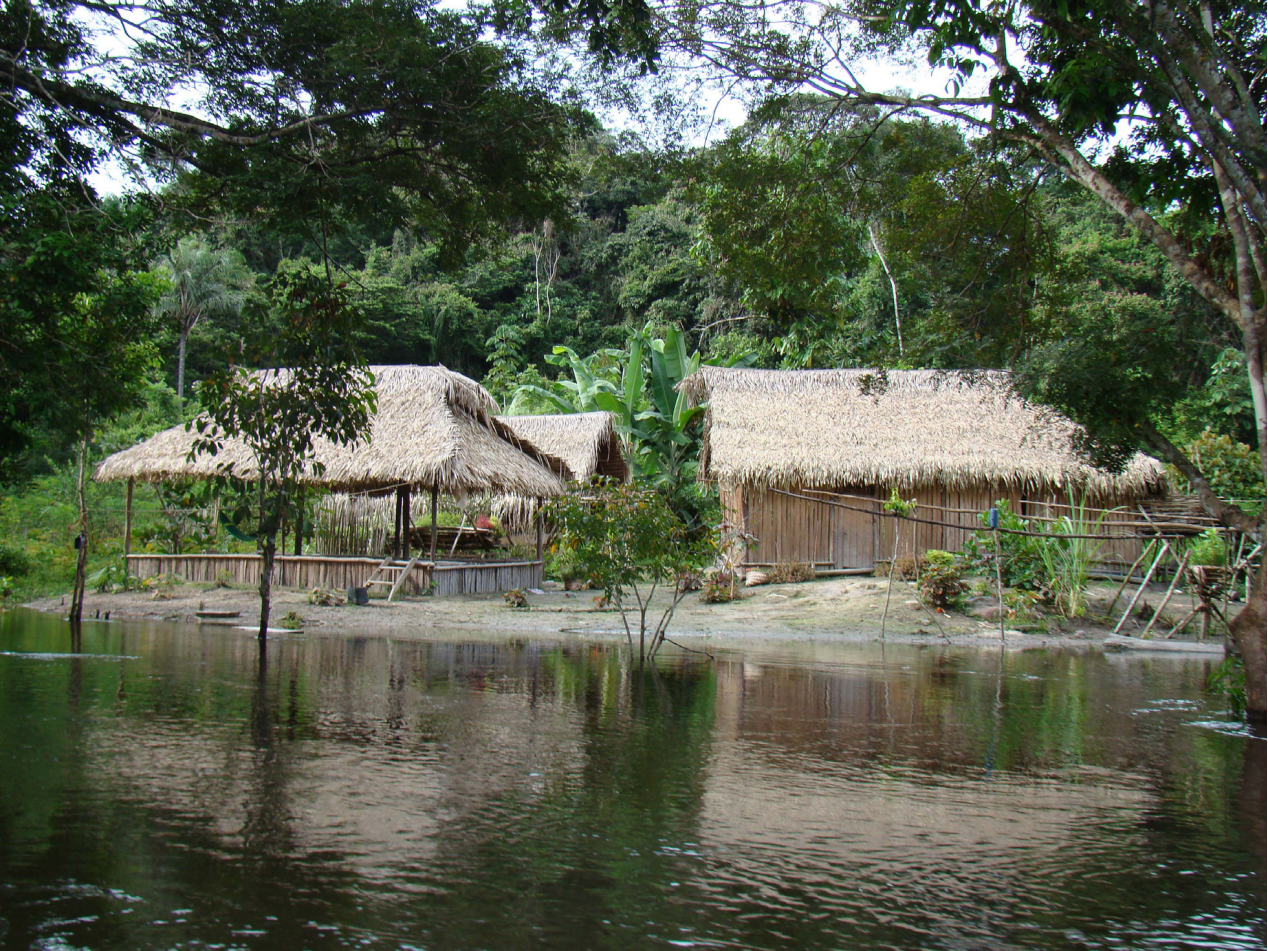
**Typical houses of the riverine population at the "Riozinho do Anfrísio Extractive Reserve". Photo by FBB**.

Currently, the local population of the "Riozinho do Anfrísio Extractive Reserve" depends primarily on the gathering of forest products such as honey, the Brazil-nut (*Bertholletia excelsa*), the assai palm (*Euterpe oleracea*), the seringa (*Hevea brasiliensis*), the crabwood (*Carapa guianensis*) and the copaiba (*Copaifera langsdorffii*), among others. These products are important for the population's subsistence at many levels, being a source of food, alternative medicines and financial revenue. The activities of hunting and fishing are also important for their survival, since they provide a rich source of animal protein. Family based farming is also part of this group of activities, with prevalence for the manioc (*Manihot esculenta*) and maize (*Zea mays*) [38]. The houses are built on the river margins (Figure 2), but distant enough from the water level at the rainy season. They are built with wood or clay and covered with the straw of the babasu palm (*Orrbignya speciosa*). In most cases, the procurement of other types of goods, such as coffee, cooking oil and sugar, happens by means of an informal trade, which is based on the direct exchange system, known locally as ***aviamento ***or ***escambo***, this is, riverines exchange their products (such as Brazil-nut, honey, fish, etc.) for other goods brought in by traders.

### Methods

This paper was produced within the scope of a research project entitled "Biodiversity, Use of Natural Resources and Ethnoconservation in the Riozinho do Anfrísio Extractive Reserve". Research permits were obtained both from the IBAMA (the Brazilian Institution for the Environment and the Renewable Natural Resources) and from the "Riozinho do Anfrísio" local community. According to the agreed licence, researchers had to assume several ethical commitments with the community, such as the communication of the study results to the population and their transcription to the local idiom (Portuguese). The work was developed under the ethnoecological approach, following the methods proposed by Huntington [39], Marques [6] and Rodrigues [40].

We used participant observation [41] and semi-structured interviews [39] to study hunting practices and the uses and knowledge of hunted animals by the local community. Participant observation [41] has been widely used in ethnobiological studies and, according to Stepp [42], can help in our understanding of the local populations' knowledge about their environment. It works with the establishment of an adequate participation and integration of the researchers within the studied groups as a way of reducing reciprocal misgiving. Researchers participate in the tasks and customs of the studied population in order to accurately observe facts, situations and behaviours that would never occur or would be distorted in the presence of strangers [43]. In semi-structured interviews, participants are guided in the discussions by the interviewer, but the direction and scope of the interview are allowed to follow the participants' train of thought. There is neither a fixed questionnaire, nor a preset limit on the time for discussions or the topics to be covered. The interviewer may have a list of topics to discuss, which can be useful for prompting further discussions when there is a lull, but the interviewer must also be prepared for unanticipated associations made by the participants [39]. Eight field trips were carried out between June of 2008 to March of 2010, with a duration each of between ten and thirty days. About thirty families were visited.

A total of 26 hunters were interviewed, from which 25 men and only one woman. Whenever possible, the 25 hunters' wives participated in the interviews as well. The age of the interviewees varied between 18 to 83 years old (average 49 years), with 84% of them being illiterate. The questions were general, always allowing an extensive dialog. We asked about which species were the most hunted, how useful they were to the community, their general knowledge about the most and least abundant species, their knowledge about the ecological and reproductive traits of those species, and so on. Tape recorders were not used to avoid inhibiting the informants. The duration of the interviews varied from 30 to 80 minutes. In many cases the interviews allowed to confirm previous observations done during riverines' activities.

**Figure 3:**
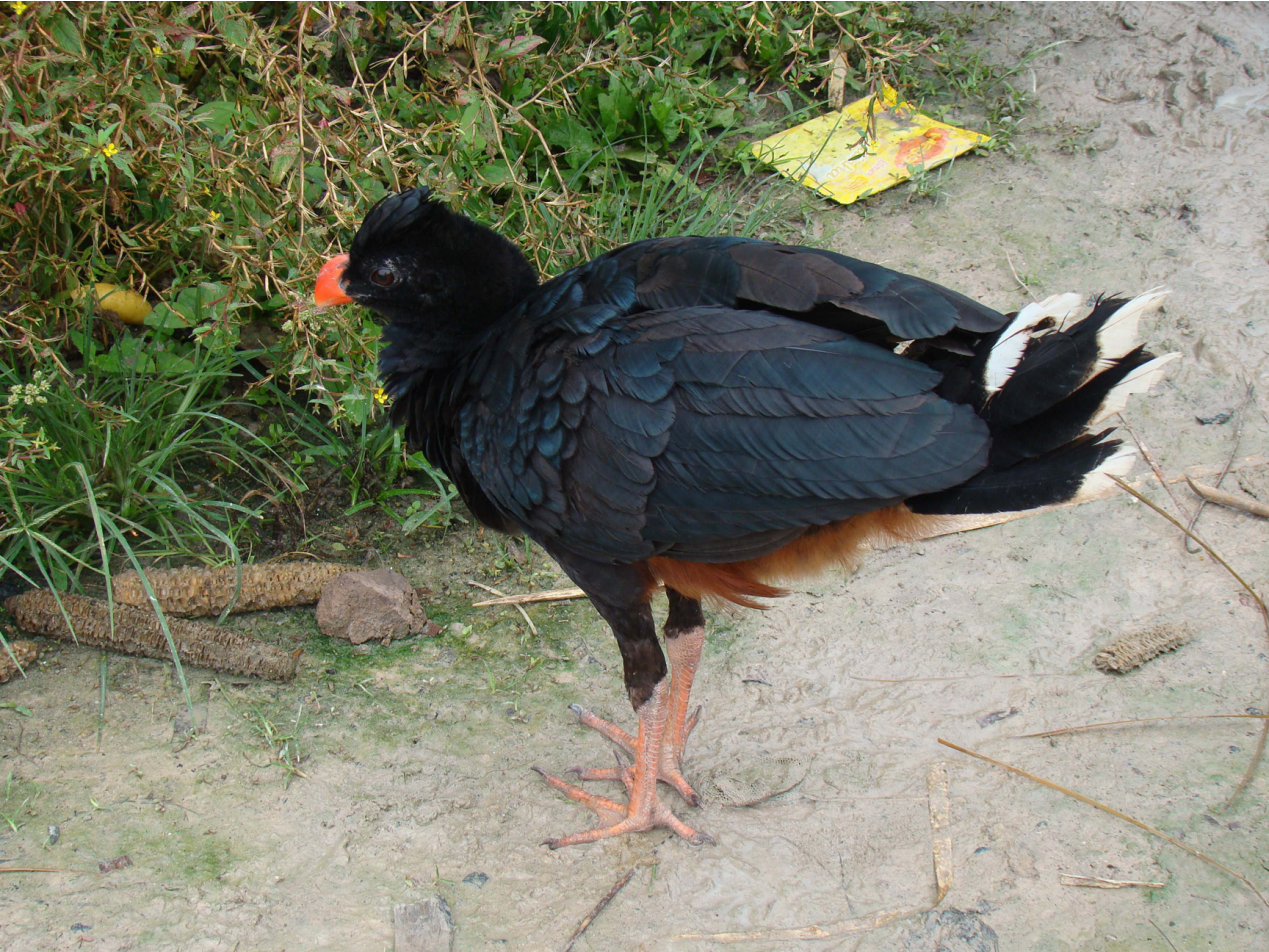
**Razor-billed curassow, *Pauxi tuberosa*.** The specimen on the photo belongs to one of the "Riozinho do Anfrísio" families, where it is raised as a pet. Photo by FBB.

Data were analysed by merging the answers of the various individuals interviewed [44]. This is, all information regarding the studied subjects was taken into account, even when it was provided by one person only. The scientific identification of the species described by the interviewees was made with the help of the relevant literature and, whenever possible, photos were taken and transferred to specialists, especially in the case of the Cracidae birds. It is important to notice that when the interviewees refer to the curassow, they can be referring to two distinct species that co-occur in the reserve: the razor-billed curassow *Pauxi tuberosa *(***mutum-fava***, Figure 3) and the bare-faced curassow *Crax fasciolata *(***mutum-pinima***). The present paper will focus on the interviewees' statements about the *P. tuberosa *because of its cultural and ecological importance, but references will be made on the *C. fasciolata *whenever appropriate.

## Results

In the "Riozinho do Anfrísio" riverine community, hunting is an activity performed mostly by men, with only two women reporting hunting activities and only in rare occasions. Based on the semi-structured interviews, all the informants (n = 26) explained that hunting is important to their lives as a source of food and, therefore, must never be forbidden by the Brazilian Environmental Institution, the IBAMA. Amongst the preferred species for hunting, the curassow appeared as one of the five most cited. The white-lipped peccary, *Tayassu pecari*, was the species that showed higher preference among the interviewees (n = 22), followed by the paca, *Cuniculus paca *(n = 8), the red brocket deer, *Mazama americana *(n = 5), the Brazilian tapir, *Tapirus terrestris *(n = 3) and the curassow (n = 3). Each interviewee named more than one preferred species.

**Figure 4:**
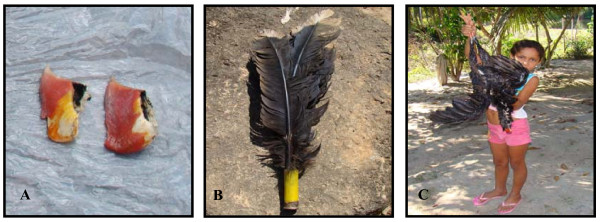
**Examples of the uses that the razor-billed curassow fulfils in the "Riozinho do Anfrísio" local community**. 4A) Two bills or "nuts" (***castanhas***), used as medicine. 4B) Tail feathers transformed into a duster (domestic tool). 4C) The hunted animal, used as food. Photos: FBB.

According to our observations and interviews, the razor-billed curassow is primarily hunted as a source of food, and its remains are used, secondarily, in traditional medicine, as a domestic tool (Figure 4), as a pet (***xerimbabo***) (see Figure 3) and as a magic-religious symbol. When eggs are found, they are collected and usually used as food, but, in alternative, they may also be preserved and hatched by chickens. The hatchlings are then raised as pets.

The hunting of curassows is, in most cases, opportunistic. It generally occurs during the hunting parties of other cynegetic species, during the gathering of Brazil-nuts (foraging parties that may take several days, with men camped in sheds away from home) and of other forest products (like lianas, honey, wood, fruits, oils, etc.), and during fishing, farming (at the corn or manioc field), etc. Occasionally, all these activities - common to the riverines' quotidian - demand considerable displacements through the forest or the river, favouring the accidental encounters of the riverines with this Cracidae species. Hunters use fire weapons to catch the bird, but are not always successful, as it was not uncommon to hear hunters reporting their encounters with the bird, but their failure in capturing it. All interviewees stated that their criterion for the choice of a prey is its size, and that they only hunt juveniles by mistake. They also report avoiding females with broods. According to the interviewees, these rules are generally used on all hunted species. Furthermore, all interviewees stated that they hunted only to feed their family and to share the harvest with their neighbors.

Although the majority of the "Riozinho do Anfrísio" population may consider the razor-billed curassow as a type of food that, in some occasions, may even be harmful (in a situation of disease, in postoperative periods, etc.), its meat is quite valued in the community, and is, most of the time, consumed boiled. We identified four common secondary uses for the razor-billed curassow.

The first is for the feathers of its tail, which are generally used for making a duster, utilized for house cleaning. In all the houses that we visited, dusters made from razor-billed curassow feathers were present. Another use of feathers, along with those of other birds, such as parrots, guans and tinamous, is as a symbolic tool for hunting rituals, in order to protect the hunters from bad luck (***panema ***in the local language). The ritual is private since the hunter must be alone and cannot be seen by anyone. The feathers are mixed and then burned by the hunter at a crossroad near the forest. During the ritual, he must receive the smoke of the burning feathers. Once the ritual is performed, he is ready to enter the forest and start hunting, but he must return home through a different path.

A third use for the razor-billed curassow is medicinal. The curassow bill, locally denominated as the nut (***castanha***), is used for the healing of "insect" and snake bites (see Table 1). It is important to note that the riverines include many venomous animals in the ethno-category "insects", including spiders, scorpions, toads, snakes, etc. Once the bird has been killed, the bill is removed, dried up and preserved. When an accident happens, with a snake for example, the dried curassow bill is used to make a tea, which is drunk by the victim. Riverines believe that the tea is an antidote to the "insect"/snake venom. The curassow gizzard is also used as medicine in order to cure various types of diseases (Table 1).

**Table 1:** The medicinal applications of the razor-billed curassow body parts, by the "Riozinho do Anfrísio" local community.

Body parts	Medical applications	Number of times reported
Bill (nut)	"Insect" and snake bites	9
	Bleeding	6
		
Gizzard	Pneumonia	1
	Bleeding	1
	Children's lack of appetite	1
	Indigestion	1
	Stroke (CVA)	1

The first column describes the body parts that are used by the community as medicines; the second column the medicinal applications of each body part; and the third column the number of times that this medicinal application was reported by the interviewees.

The fourth use for the razor-billed curassow is as a pet. Within the studied community, we found that five families were raising some type of Cracidae bird. When *P. tuberosa *brood-raising females are accidentally killed, some of the interviewees told us that they bring the chicks home to raise them as pets, as they are aware that a chick without their mother's care becomes highly vulnerable and will probably die. This behaviour is not only observed with the curassow, but also with other vertebrate species that the community usually hunts. The domesticated specimens that reside with the families as pets receive special treatments: they are raised free and carefully cared by every member of the family. Some may even cohabit with the families inside their houses, as it is the case for Psittacidae birds (parrots and parakeets), tortoises (Chelonia) and some mammals, such as white-lipped peccaries, pacas, etc. These pets are never to be killed by humans and, therefore, are not domesticated for future consumption. Moreover, when they are attacked and killed by wild animals, or when they simply disappear, the members of their adopting family express sadness. In the case of mammals, some informers reported that when they reach the reproductive maturity, they return to the forest.

Regarding the population's ethnoecological knowledge of the razor-billed curassow, 81% of the interviewees reported that this species raises two chicks at a time and that they reproduce only once each year. According to the interviewees, the species can either be seen inside the primary forest or inside shorter and more open vegetation at the river margins. The mean weights of *P. tuberosa *reported by the 25 interviewees is 3.4 kg (Table 2). The interviewees also described that the diet of *P. tuberosa *is mainly composed of fruits from the seringa tree (*Hevea brasiliensis*), the nance tree (*Byrsonima crassifolia*), the bacaba palm (*Oenocarpus bacaba*) and the assai palm (*Euterpe oleracea*), as well as worms. The interviewees reported that the curassow major predators are the margay (*Leopardus *spp.), the jaguar (*Panthera onca*), the ocelot (*Leopardus pardalis*), the puma (*Puma concolor*) and the sparrow hawks (*Accipiter *sp.).

**Table 2:** The razor-billed curassow weight according to the interviewees.

Number of interviewees (n)	Razor-billed curassow weight (kg)
1	1,5
8	2
1	2,5
6	3
4	4
2	5
2	6
1	8

N = 25 (excluding one interviewee that did not know the answer). Mean weight: 3.4 kg

## Discussion

Although generally forbidden by law in Brazil [45], hunting is practiced by many populations, mainly by those that live in rural zones. In these regions, the traditional populations, including the indigenous ones, are, in fact, allowed to hunt, but only for their subsistence. As shown in many studies, hunting has an important function in the diets of many rural populations [8,23,46-48]. This raises the question of whether people living in protected areas are beneficial or detrimental to conservation [14,49], a problem that is presently generating a large debate. In the Brazilian Amazonia, some studies suggest that local populations can be allies of the conservation process rather than threats [14]. As a matter of fact, the establishment of protected areas allowing for the presence of human populations has been progressively increasing. Some examples of categories of protected areas in Brazil that allow the presence of human populations are the National Forests, the Extractive Reserves and the Reserves for Sustainable Development.

The Cracidae family is the most threatened bird family in the American fauna, with half of the large guans and many curassows considered vulnerable or threatened [50]. Yet, the observations and the data collected in the present study suggest that in the "Riozinho do Anfrísio Extractive Reserve" the use of these faunistic resources - mainly as food, but also for domestic, medicinal and magic-religious purposes - appears to happen in a sustainable way. The riverines prefer hunting bigger species, such as the white-lipped peccary *Tayassu pecari*, which was the preferred species for 22 of the interviewees. The white-lipped peccary weights considerably more (c. 30 kg) then the razor-billed curassow, therefore, nourishing one family for a longer period. It, thus, may be a better balance between the amount of protein provided and both the physical and financial efforts made by the hunter and its family, since the practice of hunting is physically wearing and the hunting cartridges are very expensive in rural regions (in the reserve they cost, on average, three times their price in the city). According to the interviewees, the white-lipped peccaries have higher reproductive rates - one to three cubs per female and more than one litter per year - and they move around in bands of up to 30 or even 200 individuals, with the species being quoted by the interviewees as highly abundant in the reserve. The informations provided by the "Riozinho do Anfrísio" population about the weight, the reproductive rate and the social behaviour of the white-lipped peccaries are in agreement with the literature [51].

The relation between the size of the reserve (736 340 ha) and the number of residents (290 people) suggests that the hunting pressure may be relatively low. The interviewees told us that by the end of the caoutchouc epoch, in the second half of the XX^th ^century, many residents of the reserve had migrated to the cities, leading to a decrease of the local population, and a diminution of the hunting pressure on the faunistic resources of the forest. Another significant change occurred by the end of the 1970 decade, when the Brazilian government started to forbid the exploitation of wild animals for commercial purposes. At the "Riozinho do Anfrísio" region, many species were hunted for the selling of their skin, a practice that was locally known as the "cat skin" epoch, which had succeeded to the "caoutchouc" epoch [52]. Unfortunately, this type of trade still occurs in some Amazonian regions, e.g. Bolivia [11]. The protection of the Amazonian fauna given today by the Brazilian government through the creation of conservation units and of specific laws against commercial hunting seems to be effective in protecting the mammal populations, specially those of larger size, like the white-lipped peccary (*Tayassu pecari*), the Brazilian tapir (*Tapirus terrestris*) and the red brocket deer (*Mazama americana*). As a consequence, the local human populations gain more biomass for their subsistence hunting. The riverines traditional hunting practices also seem to favour the sustainable management of the curassow. Some of the "Riozinho do Anfrísio" interviewees stated that whenever it is possible to detect them, pregnant females (in the case of mammals) or females with young (in the case of both mammals and birds) are not killed. Besides, most of the interviewes also reported that they only kill the necessary quantity for the feeding of their own family, and that in most cases they offer part of the hunted game to their neighbours. This reciprocal behaviour between riverines is important for the strengthening of their friendship and confidence bonds.

The practice of rearing animals as pets is, in fact, common in rural populations, including in the indigenous ones [3,16]. The pets may be captured accidentally, when mothers with young broods are killed by mistake, or intentionally, as it usually happens with Cracidae and Psittacidae (parrots, parakeets and macaws) birds, as well as terrestrial turtles and some other species. These animals are generally captured/adopted very young or, in the case of birds, still in eggs in order to be hatched by chickens.

The use of the *P. tuberosa *feathers as a domestic tool represents another ecosystem service important to the lives of the riverines and the presence of these tools in every visited house may be an indicator of the relative abundance of the species in the reserve. Feathers of the bare-faced curassow (*Crax fasciolata*) can also be used for the same function.

According to Silva [18] and to our own findings, several body parts of the captured animals are gathered and preserved by the Amazonian riverines to be used, whenever necessary, as medicines. In fact, in the "Riozinho do Anfrísio" region, every vertebrate class is used for various purposes, including the production of medicines. In the case of the *P. tuberosa*, both the bill and the gizzard are used by the "Riozinho do Anfrísio" population for medicinal purposes. In agreement to our findings, many other studies have already registered the importance of animals in popular medicine, namely birds, where they are used to heal physical and spiritual diseases [6,9,11,53]. As in the case of *P. tuberosa*, other Cracidae birds are used by the Amazonian populations with a medicinal function. For example, the *Crax globulosa *is used in a Peruvian region to cure rheumatism and to remove the "negative energy" from people [12]. Likewise, Tejada *et al*. [11] have reported the use of the Trinidad piping-guan *Pipile pipile *and of the *P. tuberosa *as medicines among the Tucana people, in Bolivia. Silva [18] has also registered the use of feathers from several species of *Crax *to heal some diseases among the riverines of the Negro River (Amazonian basin, Brazil), including strokes. The practice of zootherapy has been studied from various perspectives [1,6,7,9,17-20] and, in the Amazonian case, is becoming a very promising field of research to both the pharmacological and biotechnology industries, as it already happen in the case of the giant leaf frog *Phyllomedusa bicolor *[53]. This is, however, a controversial subject, as very rarely the local communities have benefited from the commercialization of products and medicines by biotechnology corporations [54,55]. Recently, some hope that the knowledge of local communities could start to be more valued and rewarded came from the Conference of the Parties of the Convention on Biological Diversity meeting in Nagoya, which adopted the Protocol on Access and Benefit Sharing.

Regarding the use of animal's body parts for magic-religious purposes, such as the use of *P. tuberosa *feathers observed in this study, Tejada *et al*. [11] reported this type of practice among the Tucana people as well. These people believe that the use of the feathers and bones of the American Harpy Eagle *Harpia harpyja *(Accipitridae) in rituals will give them good luck during hunting.

Although in our study we found no evidence for the use of body parts of *P. tuberosa *for handicraft manufacturing, it is known from the literature that *C. globulosa *in Peru [12] and *P. tuberosa *in Bolivia [11] are used for the handicraft of necklaces and bracelets.

Finally, concerning the ethnoecological knowledge of the "Riozinho do Anfrísio" riverines on the razor-billed curassow, we found that it is consistent with the scientific literature (see Table 3), and consistent with the hypothesis that the Amazonian riverines hold a wide knowledge on their local biodiversity, mainly with respect to the species that are somehow used by them. It is worthwhile noticing that in Muñoz & Kattan [28], the interviewees reported that the *P. tuberosa *feeds not only on fruits and invertebrates, but also on vertebrates, like toads and caecilians, which is in agreement with the literature.

**Table 3:** Comparison between the informations of riverines and the scientific literature about P. tuberosa and others species of curassows.

Parameters	Ethnoecological Knowledge (*P. tuberosa*)	Scientific Knowledge (*P. tuberosa *and others species)
Weight (kg)	Average = 3,4 (25 interviewees)	3,5 - *Mitu tomentosa *(Souza-Mazurek et al. 2000); 3,06 - *Mitu tuberosa *[25]

Number of eggs	2 (16 interviewees)	2 to 6 eggs - *Crax globulosa *[13]

Food	Assai palm, seringa tree, bacaba palm, others fruits, worm (all interviewees)	*M. tuberosa *- Fruits (52%), leaves (8%), invertebrates (12%), vertebrates (12%) [28]

Habitat	Water-edge and interior primary forest (all interviewees)	"Riverside and forest" [30]; "...especially curassows, are associated with and dependent on pristine habitat" [26]

Social Behaviour	The curassows live in pairs or groups of up to seven individuals (all interviewees)	"Curassows were sometimes sighted in groups of two or more individuals interacting or engaging in synchronous behaviour" [30]; "*C. globulosa *live in pairs" [13]

The occurrence of *P. tuberosa *and *C. fasciolata *in the "Riozinho do Anfrísio Extractive Reserve", suggests that hunting in this region is a sustainable practice, since both species, as well other vertebrates, like the *Tapirus terrestris *and some primates of the Atelidae family [56] that also occur in the region, are bioindicators of conserved ecosystems. Future studies on the population density and habitat use of the *P. tuberosa *and other Cracidae species at the "Riozinho do Anfrísio" and other protected areas are urgent in order to reliably evaluate the actual effects of hunting on these important birds. Hill *et al*. [30] studied the population density of *C. globulosa *and *P. tuberosa *in a Bolivian region, close to a hunting area, and registered a density of 3.7 razor-billed curassows per km^2^, having estimated the occurrence of 555 individuals within an area of 150 km^2^. We do not have any field data to estimate absolute population density of *P. tuberosa *in the "Riozinho do Anfrísio Extractive Reserve", but we saw the species in five instances during the field work (132 field days), we found it being bred as a pet in four houses, and we found feathers in all houses, which suggests that the species is not extremely rare.

Many studies in various regions of the world have been showing the relevance of interdisciplinary works that value the local populations' participation, taking into account their cultural traits as a way of reconciling the nature's conservation with the presence of humans [22,57-60]. According to Hanazaki [61], conservation units like the "Extractive Reserves" are clear examples of how the local populations' ecological knowledge may be important to the nature's management and conservation. The present study is, therefore, a good example of how the local knowledge may be incorporated in management plans and conservation actions. Action projects that integrate governments, scientists, social movements, NGOs and local populations are crucial for the management success of the protected areas. It is important to understand that the involvement of the forest inhabitants may be fundamental to the whole process. Nevertheless, it is likely that conservation units of integral protection - those that do not allow the presence of human populations inside it, as is the case of the "Terra do Meio Ecological Station" and the "Serra do Pardo National Park"- are also playing an important role in the conservation of *P. tuberosa*. Along with the "Riozinho do Anfrísio Extractive Reserve", the mosaic of protected areas of the Terra do Meio Region is an interesting example of a multi-dimensional approach to conservation.

## Conclusions

The practice of hunting at the "Riozinho do Anfrísio Extractive Reserve" seems to be sustainable and apparently it has little affected the populations of Cracidae birds that occur in the region. As pointed out by the literature, these birds play an important role in the seed dispersal of forest species. The preservation of their habitats, the practice of hunting and farming for subsistence purposes only, and the controlled/diversified exploitation of other forest resources suggests that the riverines from this Amazonian region assist the conservation process of the Cracidae species, as well as of the entire forest ecosystem.

The establishment of protected areas of sustainable use that reconciles the conservation of nature with the presence of human populations is a good model for the management of nature. It is clear that the simple creation of a conservation unit doesn't mean that the natural resources within it will be automatically preserved, or even restored. It is fundamental to apply public policies and actions that would integrate the government promoters of conservation along with those that actually perform ethnoconservation *in loco*, which in the present case are the riverines. An important aspect to be taken into account, however, is the size of the human populations inside the protected regions. High birth rates may be detrimental to the sustainability of the ecosystem and, therefore, should be considered in the future as a potential problem. Yet, this is a controversial issue, since some authors disagree with the establishment of birth-rate controlling plans for the traditional populations living in areas of relevant conservation interest [62].

The razor-billed curassow, as well as other species of the "Riozinho do Anfrísio" local fauna, is an important provider of ecosystem services to the studied community, particularly as a source of food and of alternative medicines. Besides the species' intrinsic ecological value to the Amazonian forest, the provisioning of this kind of services to the human populations should be used as an additional argument to its conservation. Studies on the population ecology of Cracidae birds should be encouraged to effectively estimate the population size of these species, hence their conservation status, and to deepen our understanding of the role of Cracidae in the forest equilibrium of this region of the Oriental Amazonia.

## Competing interests

The authors declare that they have no competing interests.

## Authors' contributions

FBB was responsible for the field work and for the first draft of the manuscript. HMP and LV participated on the coordination and guidance of the research. All authors have written, read and approved the final version of the manuscript.
